# Acute effects of ionizing radiation on human endothelial barrier function

**DOI:** 10.1093/jrr/rrt171

**Published:** 2014-03

**Authors:** Peter Grabham, Preety Sharma

**Affiliations:** Center for Radiological Research, Columbia University, VC 11-205A/243, 630 West 168th Street, New York, NY 10032, USA

**Keywords:** endothelial barrier, radiation, charged particles

## Abstract

The human vasculature is critical to healthy functioning of the tissues of the body and a major factor in maintaining homeostasis is the endothelial barrier. In the brain, the blood–brain barrier (BBB) is highly specialized in order to sustain the neural tissue.

Here, we have examined the effects of radiation on BBB models using a unique variety of endpoints to assess barrier function. These include trans-endothelial electrical resistance (TEER), morphological effects, localization of adhesion and cell junction proteins (in two-dimensional monolayers and in three-dimensional tissue models) and permeability of molecules through the endothelial barrier. Two culture conditions were used to represent conditions on the inside or the lumen of vessels and conditions on the outside or ablumenal side of vessels. For the lumen, cells were cultured in serum and growth factor containing media, and for the ablumenal side of vessels, cells were cultured in serum-free defined media.

Initial experiments with gamma rays in serum-free conditions revealed a previously unknown acute effect involving cell detachment and the loss of the clinically relevant cell adhesion molecule—cell platelet endothelial adhesion molecule (PECAM)-1 [ 1]. Gamma radiation (5 Gy) induced a rapid and transient decrease in TEER at 3 h, with effects also seen at the lower radiotherapy dose of 2 Gy. This dip in resistance correlated with the transient loss PECAM-1 in discrete areas where cells often detached from the monolayer leaving gaps. Loss in PECAM-1 occurred at least in part as detached microparticles. Redistribution of PECAM-1 microparticles was also seen in three-dimensional human tissue models. By 6 h, the remaining cells had migrated to reseal the barrier, coincident with TEER returning to control levels. Resealed monolayers contained fewer cells per unit area and their barrier function was weakened as corroborated by an increased permeability over 24 h. Because PECAM-1 is involved in barrier function and platelet aggregation, this effect is likely highly relevant to cancer radiotherapy using gamma rays.

These studies were extended to include low linear energy transfer (LET) photons of X-rays and ion particles present in the space environment—low LET ion particles including high energy (1 GeV) protons and helium ions. X-rays under serum-free conditions also showed an acute response involving a dip in TEER at 3 h and the loss of PECAM-1 between cells as microparticles. Ion particles, however, did not show these effects of photons under serum-free conditions. Both protons and helium ions at doses up to 5 Gy did not produce this transient change in TEER or PECAM-1, although some longer term effects in TEER were noted.

In the presence of serum and growth factors, however, all radiations tested showed short-term effects in TEER, that of a series of symmetrical peaks which diminished in size over several hours. For 1 GeV protons and helium ions, this effect could be fitted to an equation for under-damped oscillation, a pattern typical of a mechanism for timing of events in periodic processes. For gamma and X-rays, the underdamped oscillation was present but superimposed on a drop in resistance at 3 h similar to that seen in serum-free conditions.

In conclusion, we have shown two acute effects of low LET radiation on the human endothelial barrier. First, a short-term effect of photons but not ion particles involving a single dip in TEER and the loss of PECAM-1 at 3 h after irradiation, and second an underdamped oscillation of TEER induced by both photons and ion particles that to date does not appear to be associated with the loss of PECAM-1 or any other junction molecules.
